# [Expression of Concern] AHIF promotes glioblastoma progression and radioresistance via exosomes

**DOI:** 10.3892/ijo.2026.5883

**Published:** 2026-04-15

**Authors:** Xuejun Dai, Keman Liao, Zhijun Zhuang, Binghong Chen, Zhiyi Zhou, Sunhai Zhou, Guoshi Lin, Feifei Zhang, Yingying Lin, Yifeng Miao, Zhiqiang Li, Renhua Huang, Yongming Qiu, Ruisheng Lin

Int J Oncol 54: 261-270, 2019; DOI: 10.3892/ijo.2018.4621

Following the publication of the above paper, it was drawn to the Editor's attention by a concerned reader that, in Fig. 2E on p. 264, the Transwell invasion assay results shown in the "T98G-AHIF KD" data panel appeared to potentially contain an overlapping section with the "U251-AHIF OE" data panel in Fig. 3E.

The authors were contacted by the Editorial Office to offer an explanation for this apparent anomaly in the presentation of the data in this paper; however, up to this time, no response from them has been forthcoming. Owing to the fact that the Editorial Office has been made aware of potential issues surrounding the scientific integrity of this paper, we are issuing an Expression of Concern to notify readers of this potential problem while the Editorial Office continues to investigate this matter further.

